# Randomized controlled phase III trial of adjuvant chemo-immunotherapy with activated killer T cells and dendritic cells in patients with resected primary lung cancer

**DOI:** 10.1007/s00262-014-1613-0

**Published:** 2014-09-28

**Authors:** Hideki Kimura, Yukiko Matsui, Aki Ishikawa, Takahiro Nakajima, Mitsuru Yoshino, Yuichi Sakairi

**Affiliations:** 1 Division of Thoracic Diseases, Chiba Cancer Center, Chiba, Japan; 2 Department of General Thoracic Surgery, Chiba University Graduate School of Medicine, Chiba, Japan; 3 Present Address: Saiseikai Narashino Hospital, Izumi-cho 1-1-1, Narashino City, Chiba 275-8580 Japan

**Keywords:** Phase III study, Lung cancer, Adjuvant therapy, Immunotherapy, Lymph node, Dendritic cell

## Abstract

**Purpose:**

We conducted a phase III randomized controlled trial (RCT) to investigate the efficacy of postsurgical adjuvant immunotherapy combined with chemotherapy. The immunotherapy targets were residual micrometastases and clones resistant to chemotherapy.

**Patients and methods:**

Between April 2007 and July 2012, 103 postsurgical non-small cell lung cancer patients were randomly assigned to receive either chemo-immunotherapy (group A) or chemotherapy (group B). The immunotherapy consisted of the adoptive transfer of autologous activated killer T cells and dendritic cells obtained from the lung cancer patients’ own regional lymph nodes.

**Results:**

The 2-year overall survival rates in groups A and B were 93.4 and 66.0 %, and the 5-year rates were 81.4 and 48.3 %, respectively. The differences were statistically significantly better in group A. The hazard ratio (HR) was 0.229 (*p* = 0.0013). The 2- and 5-year recurrence-free survival rates were 68.5, 41.4 and 56.8, 26.2 % in groups A and B, respectively. Those differences were also statistically significant (log-rank test *p* = 0.0020). The HR was 0.423 (*p* = 0.0027) in favor of group A. As for adverse reactions to immunotherapy, of a total of 762 courses, 52 (6.8 %) were accompanied with chills and shivering, and 47 (6.2 %), with fever (>38 °C).

**Conclusions:**

Immunotherapy has the potential to improve the postsurgical prognosis of lung cancer patients, but a large-scale multi-institutional RCT is awaited for further confirmation of this study.

**Electronic supplementary material:**

The online version of this article (doi:10.1007/s00262-014-1613-0) contains supplementary material, which is available to authorized users.

## Introduction

Lung cancer is the leading cause of cancer deaths in many advanced countries. Although molecular targeted therapy and new anticancer drugs have improved the prognosis, the overall survival rate is still only 20–30 %. Early diagnosis and surgery are the best ways to cure lung cancer, but most cases are detected at an advanced stage. Only one-third of patients are in stages I–II and become candidates for surgery. Another third receive chemotherapy and/or radiotherapy, while the rest are in far advanced stages and receive only the best supportive care. Furthermore, more than half of the patients, who are supposed to have undergone complete resection, subsequently undergo relapse in distant organs. One of the reasons for this poor prognosis may be the biological nature of lung cancer acquired during the course of cancer onset and progression. Intra-tumor heterogeneity within the primary tumors [1, 2] of lung cancer gives rise to clones resistant to chemotherapy and/or radiation therapy even if the initial response to those treatments is effective. Furthermore, a propensity for early dissemination and metastasis [3, 4] causes relapse after surgery or radiotherapy. Platinum-based doublet chemotherapy has been reported to improve the prognosis of postsurgical patients, but the impact on survival is modest and seems to have reached an efficacy plateau in the past decade [5–7].

We conducted a phase III randomized controlled study to investigate the efficacy of postsurgical adjuvant chemo-immunotherapy [8] using autologous activated killer T cells and dendritic cells (AKT-DC) [9]. Since most recurrences after surgery with adjuvant chemotherapy derive from chemotherapy-resistant micrometastases, we targeted residual micrometastases resistant to chemotherapy.

## Patients and methods

### Study design and inclusion criteria

**Fig. 1:**
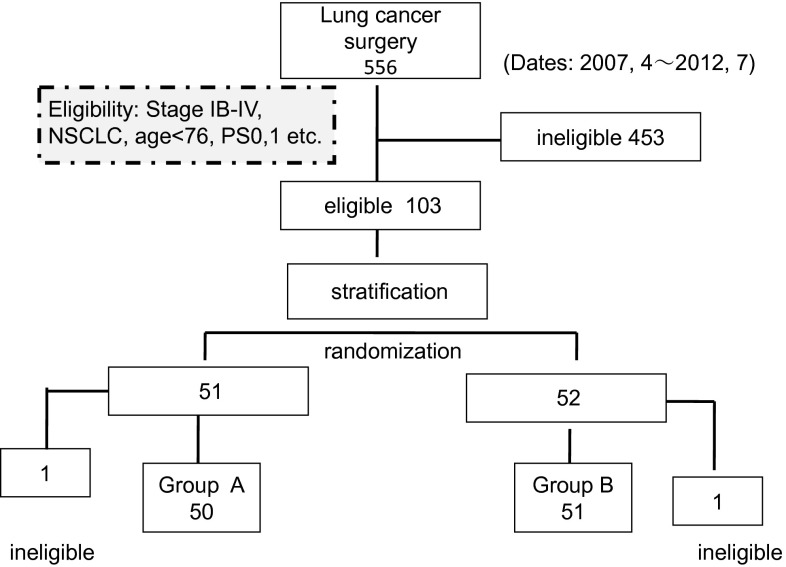
CONSORT diagram. Out of 556 cases treated surgically from April 2007 to July 2012, 103 eligible cases were randomized to receive chemo-immunotherapy (group A) or chemotherapy (group B). Ineligible cases (1 case each in groups A and B) were excluded, and 50 and 51 group A and B cases, respectively, were treated

**Fig. 2:**
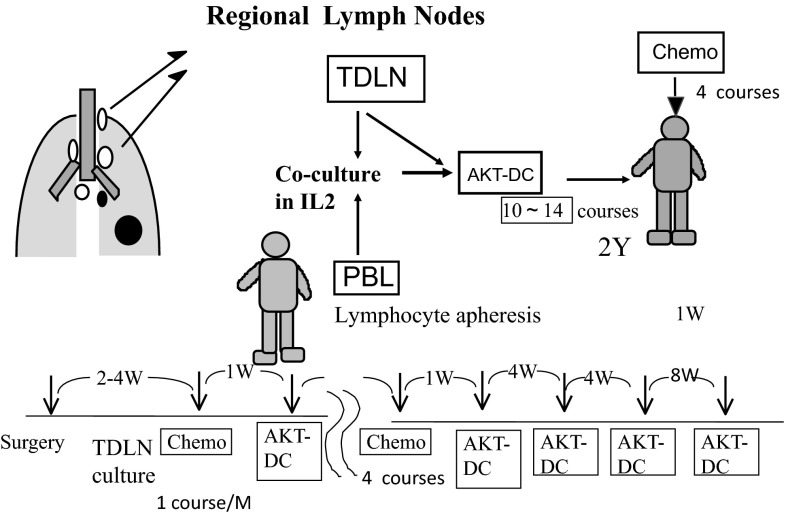
Procedure for chemo-immunotherapy. Tumor-draining regional lymph nodes (TDLN) with no metastasis were obtained at surgery, minced aseptically, and cultured in lymphocyte medium containing IL-2. Activated killer T cells and dendritic cells (AKT-DC) released from TDLN were harvested, washed, and transferred to the patients every month, beginning 1 week after adjuvant chemotherapy for 4 courses. Immunotherapy was continued every month for 6 months and then every 2 months until 2 years after surgery. When TDLN stopped releasing AKT-DC, peripheral blood lymphocytes obtained by lymphocyte apheresis were added and co-cultured with TDLN

Patients with postsurgical non-small cell lung cancer were randomly assigned to receive either adjuvant chemo-immunotherapy (immunotherapy arm: group A) or adjuvant chemotherapy (control arm: group B) (Fig. 1). Immunotherapy consisted of the adoptive transfer of AKT-DC derived from the regional lymph nodes of lung cancer patients (Fig. 2). Study inclusion criteria were as follows: postsurgical patients aged <76; Eastern Cooperative Oncology Group (ECOG) performance status (PS) 0–1; adequate bone marrow function, liver function, and renal function; histology: primary NSCLC (including combined-type small cell carcinoma); and pathological stage: IB with tumor sizes larger than 5 cm or with severe vessel invasion and stages II–IV (TNM staging system version 6). Although the indications for thoracotomy are limited to clinical stages I–II and stage IIIA after induction chemotherapy, stage IIIB and IV cases with malignant pleural effusion, micrometastasis to mediastinal lymph nodes or intrapulmonary metastasis identified after thoracotomy were also included. Non-curative resection cases were included, but exploratory thoracotomies or cases with macroscopic residual tumors were excluded. The protocol was reviewed and approved by the Ethics Committee of Chiba Cancer Center and the University Hospital Medical Information Network in Japan (UMIN: 000007525). All patients provided written informed consent. This study was conducted in accordance with the ethical principles of the Declaration of Helsinki and the International Conference on Harmonization of Good Clinical Practice guidelines.

### Other inclusion criteria

In addition to the criteria cited above, the results of in vitro examination of lymph node activity were also required for inclusion in the study. The autologous AKT-DC from the regional lymph nodes of patients had to grow enough to provide more than 7 × 10^9^ cells for each course of the therapy. Exclusion criteria for the study were as follows: a positive response to HIV, hepatitis C virus, or human T cell lymphotropic virus antibodies; a positive response to hepatitis B surface antigen; and evidence of another active malignant neoplasm.

### Randomization

Before enrolling for randomization after surgery, patients were stratified according to their stage, curability, and whether or not they received induction chemotherapy. Those who had received surgery at first were stratified by pathological stage: group I, stage IB; group II, stage II; group III, stage IIIA; and group IV, stages IIIB and IV. Those who had received induction chemotherapy were stratified to group V, stage IIIA and group VI, stages IIIB and IV. Those patients whose surgery was expiratory thoracotomy or in whom macroscopic residual tumors remained after surgery were excluded from the study. Patients with microscopic residual tumors detected after pathological examinations were included in the study, but were assigned to a more advanced stage. For example, stage IIIA patients with a positive margin of lymph nodes, a bronchial stump, or an arterial stump were stratified to group IV as stages IIIB and IV.

After all criteria had been met and written informed consent for enrollment in the study had been obtained, randomization was performed by the Internet Data and Information Center for Medical Research (INDICE) of the University Hospital Medical Information Network (UMIN) of Japan (file name: CCCI).

### Treatment

Those who were allocated to group A (immunotherapy arm) received 4 monthly courses of postsurgical chemotherapy. Adoptive immunotherapy using AKT-DC was added 1 week after each course of chemotherapy and was then continued once a month for the first 6 months after resection and then every 2 months until 2 years after surgery. This amounted to a total of 12–15 courses in 2 years (Fig. 2). Group B (control arm) received four courses of postsurgical chemotherapy. Stage IIIA patients received two courses of induction chemotherapy before surgery. Those patients underwent thoracotomy with extended lymph node dissection (ND3α) via median sternotomy [10], followed by two courses of chemotherapy.

### Chemotherapy regimens

Since no standard regimen had been established in the adjuvant setting at the time when this study was designed [11–13], we decided to use platinum doublet regimens belonging to the third-generation drugs described in Data Supplement 1.

#### Preparation of activated killer T cells and dendritic cells from regional lymph nodes

The procedure for the preparation of AKT-DC has been described elsewhere [9] (Fig. 2). Tumor-draining lymph nodes include those from the intra-pulmonary to the mediastinal lymph nodes. We used 1–2 g of regional lymph nodes located as near to the primary tumors as possible with no metastasis. If there was no metastasis, we used intra-pulmonary or hilar lymph nodes, and if metastasis had already taken place as far as the mediastinal lymph nodes, we chose mediastinal lymph nodes without metastasis.

Halves of the two or three tumor-draining regional lymph nodes (TDLN) with no tumor metastasis were rinsed with 50 ml of RPMI-1640 medium (Life Technologies Co., Japan) containing antibiotics, while their other halves were submitted for pathological examination for the presence of metastasis. Those in which no metastasis was found were transferred to a sterile Petri dish and minced aseptically into 1-mm^3^ tissue fragments. Each one showing evidence of metastasis was discarded. The tissue preparation was then suspended in 50 ml KBM-400 (Kojin Bio Co., Tokyo, Japan) or Alyse (ALyS505N: Cell Science and Technology Institute, Inc., Sendai, Japan) serum-free lymphocyte medium containing 400 IU/ml human recombinant interleukin 2 (Proleukin; Chiron B.V., Amsterdam, Netherlands), transferred to a 75-cm^2^ culture flask, and incubated at 37 °C in air containing 5 % CO_2_. When the TDLN started to release AKT-DC—usually 2–3 weeks after the initiation of the culture—the tissue and cells were transferred to a culture bag (lot 130129: NIPRO Osaka, Japan) specifically designed for lymph node tissue cultures. Half the volume of fresh medium was added every 2–3 days as long as the cells continued to proliferate exponentially. The AKT-DC generated were separated from the TDLN tissue by filtering through a nylon mesh and were then transferred to another bag. The TDLN tissue culture was continued until the propagation of the cells stopped. The AKT-DC suspension was split 2–3 times every 3–4 days into new bags each containing 800 ml of fresh medium. Then, cells containing AKT-DC were harvested, washed twice using 200 ml of saline suspended in the cryoprotective agent CP-1 (Kyokuto Pharm. Co., Tokyo, Japan) with 4 % human albumin, and stored, 5–10 × 10^9^ cells/bag (freeze bag F-100A: NIPRO Osaka, Japan) at −80 °C until used. Usually, the TDLN cultures continued to release AKT-DC for 2–3 months. When the TDLN stopped releasing cells, 1–2 × 10^9^ peripheral blood lymphocytes (PBL) obtained by lymphocyte apheresis with a COBE Spectra System (COBE BCT, Inc., Colorado, USA) were added. TDLN together with a PBL culture were carried out until we obtained a sufficient number of AKT-DC (1–3 × 10^11^ cells) for 12–14 courses of immunotherapy.

#### Certification of cells before transfer to patients

As stated elsewhere, since the cells were cultured in a sealed bag using a serum-free medium, contamination with bacteria or viruses was prevented, but all the cells underwent security tests before transfer to the patients. Cell viability was tested by Trypan blue dye exclusion methods, and cells with <90 % viability were discarded. Bacterial contamination was tested by skilled examiners with light microscopes. Endotoxin tests conducted with an endotoxin single test kit (Wako Co., Osaka, Japan) and a toxinometer (MT-353: Wako Co.), as well as culture tests, were performed whenever bacterial contamination was suspected. The cell surface markers were analyzed before initiation of the culture and just before freezing at −80 °C using two-color methods by FACS analysis with CD3, 4, 8, 25, 80, 83, B7H1, and HLA-DR monoclonal antibodies (Becton–Dickinson Biosciences, CA, USA). Cultures consisting of more than 30 % CD25+CD4+ regulatory T cells were discarded before freezing. Tumor cell contamination was tested by cytological examination with Papanicolaou stain and by immunohistochemical analysis with cytokeratin (Dako Japan Inc., Tokyo Japan) or thyroid transcription factor-1 (TTF-1 Dako) antibodies.

### Transfer to patients

Cells qualified by safety examination, and stored at −80 °C, were thawed in a 37 °C water bath and transferred to the patients intravenously with 50 ml saline.

### End points

The primary end point of this study was overall survival. Secondary end points were recurrence-free survival, toxicity, and adverse effects of immunotherapy. All eligible patients were included in the analysis of overall survival and progression-free survival.

### Follow-up

Patients received regular checkups with tumor markers (CEA, CA-199, SCC, etc.) and chest X-rays every month for the first 6 months, every 2–3 months until 2 years had passed, and every 4–6 months thereafter until 5 years after the thoracotomy. Chest CTs were performed 3, 6, and 12 months after surgery and every 4–6 months thereafter until the 5-year point. One, two, and five years after surgery and whenever recurrence was suspected, PET-CT or a bone scan and/or a brain MRI was performed.

### Diagnosis and treatment after recurrence

Recurrence was diagnosed by the Cancer Board of Thoracic Diseases in our center following the preset requirements: (1) histological or cytological evidence of recurrence discovered by biopsy and (2) tumor marker elevation or growth of new lesions accompanied by positive PET findings. Temporary elevation of tumor markers or new lesions with no apparent growth was not considered to show recurrence.

Chemotherapy was used after recurrence, and brain metastasis was treated with a gamma knife or by whole-brain irradiation. Bone or lymph node metastasis was treated by radiation. Immunotherapy was continued or resumed with the patient’s consent in combination with the chemotherapy. EGFR-mutation-positive patients received EGFR-TKI and ALK fusion gene-positive patients received ALK-TKI.

### Sample size

Pretrial assumption of sample size was made according to the following formula [14]:

$$ N = \left[ {\varepsilon / 2- {\text{S1}} - {\text{S}}0} \right]/\left( { 1- \omega } \right) $$

$$ \varepsilon = \left[ {\left( {\theta + 1} \right)/\left( {\theta - 1} \right)} \right]^{ 2} \left( {{\text{Z}}_{{\upalpha/ 2}} + {\text{Z}}_{\upbeta} } \right)^{ 2} $$

$$ \theta = { \log }\left( {\text{S1}} \right)/{ \log }\left( {{\text{S}}0} \right) $$

where S1 is the rate of survival of group A at 5 years after surgery, and S0 is the rate of survival of group B at 5 years. *ω* represents the rate of dropout and Z_α/2_ + Z_β_ = 2.8018 (from the table of normal distribution). We have set the two-sided significance level at 5 % with the power of the test at 80 %.

From the phase II study conducted between 1998 and 2004, it was estimated that 45 patients would be required per arm of the study. Taking into account the possibility of more dropout and death cases, we planned to enroll 60 cases per arm for 5 years.

### Statistical analysis

Statistical analysis was performed on all randomly assigned eligible patients. Overall survival was defined as the time from random assignment to the date of death from any cause. Recurrence-free survival was defined as the time from randomization until the confirmation of recurrence by our Cancer Board. Survival curves were estimated by the Kaplan–Meier technique. Duration of survival was compared between the treatment arms using a two-sided log-rank test. All data analyses were performed using Statistical Analysis Software version 9.3 (Statcom Co., Ltd., Tokyo, Japan) by the Translational Research Informatics Center (TRI) of the Ministry of Education, Culture, Sports, Science and Technology of Japan, and by the Foundation for Biomedical Research and Innovation in Kobe City. Interim analysis was scheduled for 5 years after the initiation of the study, regardless of the time of enrollment.

## Results

The study opened on April 1, 2007 and closed on July 30, 2012. Of a total of 556 patients who underwent surgery for lung cancer in our center, 103 cases were enrolled in the study, and 453 cases were ineligible and excluded from it: 110 patients were over 76 years old, 226 cases were stage IA, 46 cases were stage IB with tumor sizes of <5 cm, 35 were stage IIIB, IV with macroscopic residual tumors remaining after surgery and failed to provide enough T cells, 29 had a PS of ≧2, and 7 patients refused randomization (Fig. 1). The demographic characteristics of the patients were evenly distributed between the two groups (Table 1). Projected accrual was 120 cases, but enrollment was stopped at 103 cases in July 2012 on the basis of interim analyses conducted after 5 years. Significant survival benefit was observed in group A, and we felt that further continuation of the study with the group B control arm would be unethical. The median follow-up time was 32.2 months. Among the patients enrolled for the study, two were found to be ineligible after randomization and were finally excluded from the study.

**Table 1:** Baseline patient demographic and clinical characteristics

Characteristics	group A	Group B	*p*
Age (mean ± SD)	63.2 ± 8.1	64.5 ± 6.9	0.4709
Range	39–75	41–74	
*Sex*			
Male	37	38	
Female	13	13	0.9571
*Stage*			
IB	7	6	
II	8	7	
IIIA	22	24	0.7251
IIIB	8	11	
IV	5	3	
*T factor*			
T1	11	11	
T2	23	23	0.9253
T3	9	10	
T4	7	7	
*N factor*			
N0	16	12	
N1	8	10	0.5142
N2	23	26	
N3	3	3	
*M factor*			
M0	45	48	0.6824
M1	5	3	
*PS*			
0	44	44	
1	6	7	
*Histology*			
Ad	38	35	
Sq	6	11	
Large	2	2	0.492
Pleo	2	1	
Others	2	2	
*Stratification*			
Group I	7	6	
Group II	7	6	
Group III	13	14	
Group IV	11	10	0.5844
Group V	7	9	
Group VI	5	6	
Total	50	51	

*Ad* Adenocarcinoma, *Sq* Squamous cell carcinoma, *Large* Large-cell carcinoma, *Pleo* Pleomorphic carcinoma

### Surgery and chemotherapy

Induction chemotherapy was administered in 12 and 15 clinical stage IIIA cases in groups A and B, respectively. The types of agents administered in this chemotherapy, as well as the surgical procedures, were well balanced in the two arms, and there was no statistical difference between the groups in the numbers of courses of chemotherapy. The chemotherapy regimens of both groups are stated in DS1. The mean total numbers of courses of chemotherapy including those after recurrences in groups A and B were 5.80 ± 3.83 (SD) and 6.41 ± 6.65 (SD), respectively. EGFR-TKI was given to five patients in group A and to seven in group B after recurrences. Eight cases in group A and 11 in group B received bevacizumab in combination with or without chemotherapy. One patient in group A received an ALK fusion gene inhibitor.

### Adoptive immunotherapy

A total of 762 courses were administered to group A patients from April 2007 to February 2013. The mean number of effector cells in each course was 10.2 ± 3.1 (standard deviation; SD) × 10^9^ cells, the mean number of courses for a patient was 15.3 ± 6.92 (SD) courses, and the mean total of effector cells delivered was 1.51 ± 0.68 (SD) × 10^11^ cells. There was no correlation between the number of courses delivered to a patient and either recurrence or survival. The courses and the numbers of cells delivered to each patient are detailed in DS2.

### Adverse reactions to immunotherapy

Out of a total of 762 courses, 52 (6.8 %) were accompanied with chills and shivering, and 47 courses (6.2 %) were followed by fever (>38). Of 50 patients treated with immunotherapy, 28 had no adverse reactions and 22 had at least one adverse reaction of chills, shivering and/or fever. Chills and shivering started about 30 min after the start of each cell transfer, continued for 10–20 min, and were followed by fever up to 38–40 °C. These fevers lasted for 2–3 h, and the body temperature then gradually declined to a normal level within the same day. In no case did fever persist until the next day. No adverse reaction other than chills or fever was observed. Adverse reactions are listed in DS2 (Data Supplement 2).

### Recurrence

There were 19 cases of recurrence in group A (lungs, 6; lymph nodes, 5; bones, 3; and others, 5) and 33 in group B (lungs, 9; lymph nodes, 8; bones, 8; brain, 5; and others, 3). Seven patients in group A became tumor free after recurrence following treatments combined with immunotherapy: two cases of EGFR-TKI, three cases of resection of lung metastasis, one case of ALK fusion gene inhibitor, and 1 of radiation; and continued to be tumor free until the time of the final analysis. In one case in group B, CR was attained after chemotherapy. Therefore, 38 patients in group A and 19 patients in group B were tumor free at the time of analysis. Recurrence was seen in 14 and 21 cases of adenocarcinoma and in 3 and 7 squamous cell carcinoma cases in groups A and B, respectively. There was no correlation between recurrence and histological types.

### Survival

**Fig. 3:**
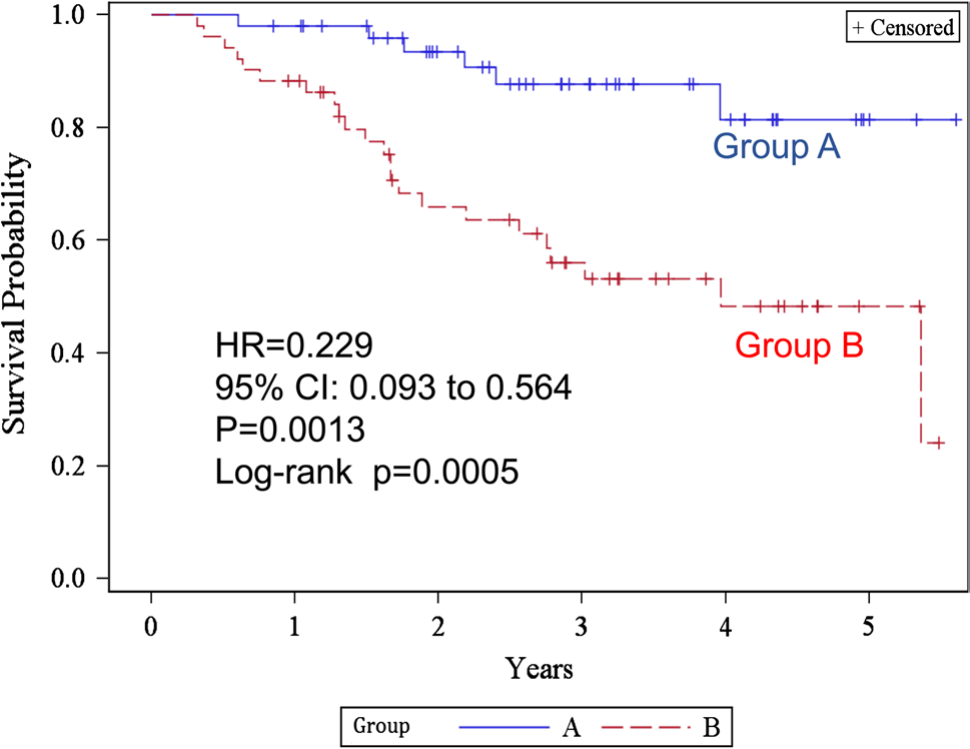
Kaplan–Meier estimates of overall survival for groups A and B

**Fig. 4:**
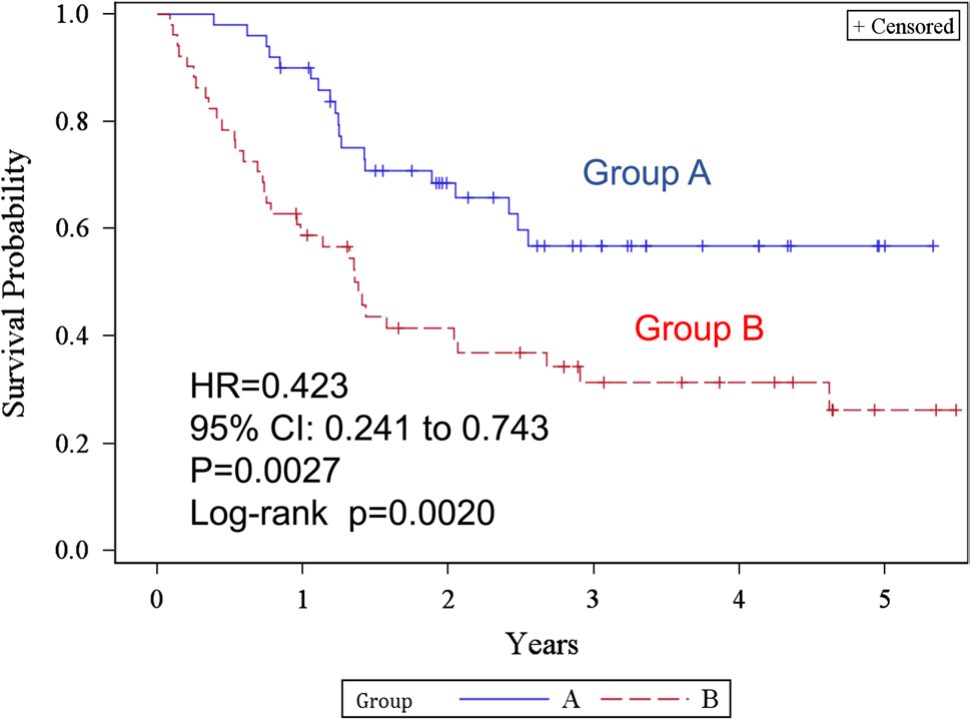
Kaplan–Meier estimates of recurrence-free survival for groups A and B

Figure 3 shows the Kaplan–Meier estimates of overall survival. The 2- and 5-year overall survival rates were 93.4 % [95 % confidence interval (CI) 80.8–97.8] and 81.4 % (60.1–92.1) in group A, and 66.0 % (50.4–77.7) and 48.3 % (31.4–63.3) in group B, respectively. The difference was statistically significant (log-rank test *p* = 0.0005, generalized Wilcoxon test *p* = 0.0005) in group A. The HR was 0.229 (95 % CI 0.093–0.564, *p* = 0.0013). The median survival time of group B was 47.5 (from 26.3 to not reached) months and was never reached in group A. The 2- and 5-year recurrence-free survival rates (Fig. 4) were 68.5 % (53.2–79.7) and 56.8 % (40.3–70.3) in group A, 41.4 % (27.5–54.7) and 26.2 % (13.1–41.5) in group B, respectively. The difference was statistically significant (log-rank test *p* = 0.0020). HR was 0.423 (95 % CI 0.241–0.743, *p* = 0.0027) in group A. The median recurrence-free survival was 16.56 (9.00–32.01) months in group B, but was not reached in group A.

## Discussion

We have reported the results of a phase III randomized controlled study of postsurgical adjuvant immunotherapy with lymphokine-activated killer (LAK) cells conducted between 1986 and 1992 [15]. The in vitro study using regional lymph nodes as a source of killer and dendritic cells indicated a much higher activity against autologous tumor cells than that using LAK cells [9]. A phase II study was conducted between 1998 and 2004 using AKT-DC obtained from the regional lymph nodes of primary lung cancer patients [16]. The results of the phase II study predicted a promising outcome for a phase III study using this modality. Furthermore, this modality yielded a more marked effect in early stage than in advanced-stage cases. Patients with macroscopic residual tumors, such as those with pleural dissemination, or bulky N2–N3 metastases, failed to respond to this treatment. Therefore, we added stage II cases and IB cases with tumor sizes larger than 5 cm to, and excluded cases with macroscopic residual tumors from, the present phase III study.

This phase III study, conducted between 2007 and 2012, is the concluding portion of a series of studies of adjuvant immunotherapy for postsurgical lung cancer patients that lasted from 1986 to 2012. In a clinical study conducted to verify the effects of chemotherapy, placebo groups are included. But in adjuvant settings, control arms do not usually use placebo or blinded procedures, but observation only. The reasons for this originate from the fact that there has been no standard chemotherapy recommended to improve survival in adjuvant settings. In this adjuvant immunotherapy, we thought that blind control with no cell infusion would be deceptive and unethical. Furthermore, there are no cells which have no effect at all—whether favorable or unfavorable—on the immune response.

The successful outcome of this study depended largely on the improvements of the in vitro system, which enabled us to obtain a large quantity of high-quality effector cells for use in each course of treatment. Long-term tissue cultures of lymph nodes in specifically designed culture bags enabled us to transfer 1–2 × 10^10^ cells for a course of treatment in a contamination-free environment. The most effective dose for immunotherapy remains a matter of conjecture, but the number of lymphocytes and dendritic cells transferred to the patients, and their activity against cancer cells appear to be the key issues that determine whether the immunotherapy will work or not. We obtained sufficient numbers of lymphocytes from N0, N1 patients, but it was difficult to do so from some of the N2, N3 patients. In half of the N2, N3 patients, usually those with tumors that were macroscopically residual, it was difficult to obtain sufficient lymphocytes. Among our 62 stage IIIB and IV cases, 35 (56.5 %) were excluded from the study because macroscopic residual tumors remained after surgery, and we could not obtain enough T cells. The cell surface markers of AKT-DC were CD3 (94.7 %), CD4 (57.9 %), CD8 (55.1 %) and CD83 (21.5 %) [9]. Although we have not evaluated precisely the correlation between cell surface markers and the effects of immunotherapy, we could not detect any relationship between cell surface markers and the results of immunotherapy so far observed.

It has been suggested that immunocompetent cells eliminate or suppress the proliferation of nascent transforming cells in the initiation of tumor growth (known as the immunoediting: elimination phase) [17–19]. However, immune selective pressure favors the growth of tumor cell clones with a low-immunogenic phenotype, and this leads to the equilibrium phase of immunoediting. In this phase, tumor cell generation and apoptosis are equivalent and keep the tumor size unchanged in an equilibrium in which tumors remain occult and asymptomatic for a prolonged period of time [20–22]. In the escape phase, when tumors start proliferating, expanding in size, and forming clinically detectable masses, the immune system, in turn, becomes inefficient or tolerates tumor growth. Numerous mechanisms have been proposed to explain how tumors escape immune control and find a way from dormancy to progression [23–26]. Immunosuppression within the tumor microenvironment has been cited as one of the causes of the ineffectiveness of immunotherapy against cancers [27–30].

The target of the immunotherapy described here is not the primary lesion, but the undetectable tumor cells remaining after the resection of a primary carcinoma [3, 4]. A major cause of tumor recurrence is metastasis that is considered to be derived from circulating and disseminating tumor cells (CDTC). Primary cancers start releasing tumor cells at relatively early stages of tumor development. CDTC released from the primary lesion remain dormant and in a quiescent state for a prolonged period of time as solitary tumor cells or dormant micrometastases [20–22]. A controversial discussion of the main reasons for immune escape mechanisms concerns whether tumor growth depends on the poor immunogenicity of tumor cells, which helps them to escape immune recognition, or on the immunosuppressive mechanisms working within the tumor microenvironment. In other words, either (1) since the tumor cells have already undergone immune surveillance, they are not recognized by the immune system and only the non-immunogenic tumor cells survive after tumor progression, or (2) tumor cells are recognized, but immune suppression in the tumor microenvironment blocks immune attack by killer cells. The results of this study suggest the importance of the latter mechanisms of the tumor microenvironment in immune escape. Immunotherapy using this modality therefore has a role in recurrence control by inhibiting the growth of disseminated micrometastases before an immunosuppressive microenvironment has been achieved.

The majority of the causes of cancer recurrence derive from CDTC, which are clinically undetectable at the time of surgery. The phenotypic diversity of disseminated cells resulting from intra-tumor heterogeneity [1, 2] gives rise to clones resistant to chemotherapy and prevents tumor cell eradication by chemotherapy. The heterogeneity of tumor cells enables them to escape even from molecular targeted therapy [31]. The regional lymph nodes of lung cancer patients are the sites where the first adoptive immune response against cancer develops [32, 33]. Dendritic cells at the tumor site take up antigens, migrate to lymph nodes, and educate antigen-specific naïve T lymphocytes to become cytotoxic T lymphocytes [34]. Using these lymph nodes as a source of dendritic cells and killer cells, we can eradicate the heterogeneous tumor cells disseminating throughout the body carrying a wide variety of antigens.

Our long experience of in vitro cultures of regional lymph nodes [35–37] shows that, although in vitro culture of non- or micrometastatic regional lymph nodes induces AKT-DC in the presence of low-dose IL2, macroscopic metastatic lymph nodes do not propagate those cells in vitro. In other words, tumor cells and lymphocytes are mutually exclusive and never coexist in vitro when cultured in the presence of IL2 for more than 2–3 weeks. Therefore, contamination of tumor cells in AKT-DC is prohibited when lymphocyte growth is dominant. This in vitro phenomenon does not seem to depend on the patient or the tumor cell types, but on the reciprocal quantitative balance between tumor cells and lymphocytes. This phenomenon indirectly provides evidence that tumor cells are recognized even after tumor progression, but the tumor microenvironment blocks the effect of immune responses.

The outcome of the present study was successful, and this may be attributable to the mechanism whereby the target of immunotherapy is the dormant CDTC remaining after surgery before an immunosuppressive microenvironment is developed. Although the results of this study have great significance, this single-institutional small-sample study needs further confirmation with a large-scale multi-institutional RCT before the clinical importance of this modality is fully recognized.

## Electronic supplementary material

Below is the link to the electronic supplementary material.

Supplementary material 1 (PDF 202 kb)

## Abbreviations

AKT-DC: Activated killer T cells and dendritic cells
ALK: Anaplastic lymphoma kinase
CDTC: Circulating and disseminating tumor cells
CI: Confidence interval
CR: Complete response
EGFR: Epidermal growth factor receptor
HR: Hazard ratio
LAK: Lymphokine-activated killer T cells
NSCLC: Non-small cell lung cancer
PR: Partial response
TDLN: Tumor-draining regional lymph nodes
TKI: Tyrosine kinase inhibitor
