# Supplementary material for: Randomized controlled phase III trial of adjuvant chemo-immunotherapy with activated killer T cells and dendritic cells in patients with resected primary lung cancer
Source: Cancer Immunol Immunother. 2014 Sep 28;64(1):51–9. doi: 10.1007/s00262-014-1613-0 (PMC4282697; doi:10.1007/s00262-014-1613-0)
Supplement: Supplementary file 1 — Supplementary material 1 (PDF 202 kb) [file 262_2014_1613_MOESM1_ESM.pdf]

Data Supplement 1

Chemotherapy Regimens and Courses for Each Patient

	Regimens		Regimens
A	CBDCA+GEM	L	bevacizumab
B	CBDCA+PTX	M	VNR
C	DOC	N	intra-thoracic CDDP
D	CBDCA+PEM	O	CBDCA+DOC
E	PEM	P	CDDP+PEM
F	CBDCA+PTX+bevacizumab		
G	CDDP+TS1(induction)		
H	CDDP+VP16		
J	CDDP+CPT11		
K	CDDP+VNR		

CBDCA: carboplatin; GEM: gemcitabine; PTX: paclitaxel; DOC: docetaxel;
PEM: pemetrexed; CDDP: cisplatin; TS-1: tegafur gimeracil oteracil potassium;
VP16: etoposide; CPT11: irinotecan; VNR: vinorelbine.

Group A

Patient No.	No of courses	Oral administration	Infusion			
A-001	3		B(X2)	A		
A-002	11	EGFR-TKI	B(X3)	A(X4)	D(x4)	
A-003	4		A(X2)	C(X2)		
A-004	12		A(X2)	C(X2)	B(X4)	J(X4)
A-005	16		H(X5)	B(X2)	C(X5)	D(X4)
A-006	2		A(2)			
A-007	9		A(X2)	C(X4)	J	E(X2)
A-008	5		B	A(X4)		
A-009	12		A(X2)	C(X2)	B(X4)	D(X4)
A-010	4		A(X4)			
A-011	12		A(X4)	C(X2)	D(X3)	F(X3)
A-012	4		B(X2)	A(X2)		
A-013	6		A(X2)	C(X2)	B(X2)	
A-014	7	EGFR-TKI	A(X2)	B(X2)	C(X3)	

A-015	0						
A-016	2		A(X2)				
A-017	11		A(X2)	C(X2)	B(X2)	D(X5)	
A-018	8		A(X4)	D(X4)			
A-019	4		A(X4)				
A-020	4	ALK-TKI	G(X2)	A(X2)			
A-021	0						
A-022	7		A(X4)	D(X3)			
A-023	7		G(X2)	A(X4)	D		
A-024	4		A(X4)				
A-025	4		A(X4)				
A-026	13	EGFR-TKI	G	A(X4)	C(X2)	D(X4)	F(X2)
A-027	4		A(X4)				
A-028	7		N(X3)	A(X4)			
A-029	13		N(X5)	A(X4)	D(X4)		
A-030	13	TKI,TS1	A(X2)	K(X4)	C(X2)	F(X2)	B(X3)
A-031	4		A(X4)				
A-032	4		A(X4)				
A-033	4		A(X4)				
A-034	4		A(X4)				
A-035	1	UFT	Q				
A-036	6	EGFR-TKI	A(X4)	D(X2)			
A-037	7		A(X4)	F	C(X2)		
A-038	4		A(X4)				
A-040	10		N(X5)	A(X2)	B(X3)		
A-041	3		A(X3)				
A-042	4		A(X4)				
A-043	0						
A-044	4		A(X4)				
A-045	4		A(X4)				
A-046	4		F(X4)				
A-047	5		F(X2)	B(X3)			
A-048	4		F(X4)				
A-049	5		B	F(X4)			
A-050	2		A(2)				
A-051	3		B	F(X2)			

Mean number of courses	5.80 (SD: 3.83)
Total number of courses	290
min	0
max	16

A-039 was found to be ineligible after randomization and was excluded from the study.

Group B

Patient No.	No of Courses	Oral administration	Infusion					
B-001	22		B(X2)	C(X2)		D(X8)	E(X7)	D(X3)
B-002	4		B(X2)	C(X2)				
B-003	1		B					
B-004	4		B(X2)	C(X2)				
B-005	0							
B-006	1		A					
B-007	3		N(X3)					
B-008	0	UFT						
B-009	0							
B-010	5		A	C(X4)				
B-011	4		A(X4)					
B-012	5	EGFR-TKI	B(X4)	B				
B-013	26		N(X3)	A(X4)	K(X4)	F(X6)	L(X6)	E(X3)
B-014	0							
B-015	4	EGFR-TKI	K(X4)					
B-016	4		A(X4)					
B-017	24	EGFR-TKI	A(X2)	O(X4)	P(X4)	E(X5)	F(X4)	L(X5)
B-018	5		A(X4)	E				
B-019	13	EGFR-TKI	A(X4)	O(X5)		D(X4)		
B-020	5		H(X5)					
B-021	6		N(X3)	A(X2)		C		
B-022	0							
B-023	8		A(X4)	F(X4)				
B-024	8		A(X4)	B(X4)				
B-025	6	EGFR-TKI	G(X2)	A(X4)				

B-026	4	EGFR-TKI	K(X4)					
B-027	28		O K(X2)	A(X4)		F(X4)	L(X13)	F(X4)
B-028	1		N					
B-029	7		A(X4)	O(X2)		C		
B-030	2		A(X2)					
B-031	4		A(X4)					
B-032	12		A(X4)	D(X4)		F(X4)		
B-033	13		A(X2)	K(X5)		F(X3)	E(X3)	
B-034	4	TS1	G(X4)					
B-035	20	EGFR-TKI	A(X2)	D(X6)	E(X4)	K	F(X3)	L(X4)
B-036	9		N(X2)	F(X7)				
B-037	1		F					
B-038	4		A(X4)					
B-039	7		A(X7)					
B-040	5		G	A(X2)		C(X2)		
B-041	0							
B-042	4		B(X4)					
B-043	6		A(X4)	C(X2)				
B-044	4		A(X4)					
B-045	4	TS1	K(X4)					
B-046	5		G(X2)	A(X3)				
B-047	7		B(X6)	C				
B-048	5		F(X2)	B		F(X2)		
B-049	6		A(X6)					
B-050	3		A	F(X2)				
B-052	4		A(X4)					

Mean number of courses	6.41	(SD:6.65)
Total number of courses	327	
min	0	
max	28	

Oral administration is not included in the calculation of the courses.

B-051 was found to be ineligible after randomization and was excluded from the study.

Data Supplement 2

Immunotherapy

Patient No.	Courses	Transferred Cell Numbers ($X10^9$) Mean / Course	Total	Adverse Effects Chills, Shivering	Fever >38°	Recurrence
A-001	3	7.1	21.3	0	0	yes
A-002	36	9.1	327.6	3	6	yes
A-003	13	9.3	120.9	0	0	no
A-004	20	9.6	192	1	0	yes
A-005	30	9.7	291	1	6	no
A-006	14	10.4	145.6	0	0	no
A-007	17	9.2	156.4	1	1	yes
A-008	14	9.1	127.4	0	0	no
A-009	31	10.9	348.9	4	0	yes
A-010	16	9.3	148.8	3	3	no
A-011	31	9.8	303.8	0	0	yes
A-012	18	9.5	171	3	4	no
A-013	16	8.8	140.8	0	0	yes
A-014	24	10.3	247.2	9	2	yes
A-015	14	11.5	161	0	6	no
A-016	14	5.6	78.4	0	0	no
A-017	24	6.6	158.4	0	0	yes
A-018	12	5.7	67.9	3	3	yes
A-019	16	8.8	140.8	4	1	no
A-020	14	8.9	124.6	2	0	yes
A-021	10	6.5	65	0	0	yes
A-022	18	7.4	133.2	1	1	no
A-023	25	7.5	187.5	0	0	no
A-024	14	7.8	109.2	0	0	no
A-025	14	5.7	79.8	0	0	no
A-026	22	10	220	1	1	yes
A-027	13	10.3	133.9	0	0	no

A-028	19	9.2	174.8	6	4	no
A-029	16	6.9	110.4	2	2	no
A-030	15	7	104.7	0	0	yes
A-031	13	7.1	92.3	0	0	no
A-032	12	8.2	98.4	0	0	no
A-033	13	12.7	165.1	0	0	no
A-034	13	10.1	131.3	0	0	yes
A-035	18	13	234	2	2	yes
A-036	14	10.2	142.8	1	0	no
A-037	14	11.5	161	1	1	yes
A-038	14	11.2	156.8	0	0	no
A-040	14	12.3	172.2	1	1	no
A-041	5	10	50	2	1	no
A-042	13	13.6	176.8	1	2	no
A-043	6	7.9	47.4	0	0	no
A-044	12	12.2	146.4	0	0	no
A-045	9	12.2	109.4	0	0	yes
A-046	11	17.7	194.7	0	0	no
A-047	11	15.3	168.3	0	0	no
A-048	10	16.3	163	0	0	no
A-049	9	19.4	174.6	0	0	yes
A-050	8	15.4	123.2	0	0	no
A-051	1	16	16	0	0	no
Mean	15.3	10.2	151.6			
SD	6.92	3.13	68.11			
Max	36	15.1	327.6			
Min	1	5.6	21.3			

Mean total of courses transferred / patient	15.3±6.92
Mean cell number transferred /course	10.2±3.13X10^9
Mean total cell number / patient	151.6±68.1X10^9
Total number of courses	762
Chills, shivering	52 (6.8%)
Fever	47 (6.2%)
